# Detection of CRISPR-Cas-induced mutations in *Daphnia*

**DOI:** 10.1093/g3journal/jkag050

**Published:** 2026-02-24

**Authors:** Swatantra Neupane, Michael E Pfrender, Li Wang, Sen Xu

**Affiliations:** Division of Biological Sciences, University of Missouri, Columbia, MO 65211, USA; Department of Biological Sciences, University of Notre Dame, Notre Dame, IN 46556, USA; Division of Biological Sciences, University of Missouri, Columbia, MO 65211, USA; Division of Biological Sciences, University of Missouri, Columbia, MO 65211, USA

**Keywords:** fragment analysis, T7 endonuclease, mutants, *Daphnia*, screening

## Abstract

CRISPR-Cas9 has established itself as a robust tool for conducting loss-of-function gene research in emerging model species, including the freshwater zooplankton *Daphnia*. However, sensitive detection of mutations, especially in genetic mosaic and pooled samples, remains a challenge. In this study, we evaluate 2 of the most widely used mutation screening techniques, the T7 Endonuclease I assay and fragment analysis for their sensitivity, accuracy, and practical use in detecting CRISPR-induced indels in 4 targeted genes, DNMT3A, DNMT3B, PERIOD2, and DMRT1 in *Daphnia magna*. Here, we show that T7 Endonuclease I, although it offers a quick and cost-effective screening method, often produces false positives, especially when examining pooled samples. Conversely, fragment analysis facilitates detecting allele size differences at a fine resolution, reproducibility in detecting indels, and distinguishing zygosity, and is more reliable as a method to detect mutation. Our comparative analyses convey the importance of carefully selecting the appropriate screening methods depending on research questions.

## Introduction

The CRISPR-Cas gene-editing system has become a leading method for generating targeted genetic modifications in various animal models (Hsu et al. 2014; Lee et al. 2020). Cas-induced DNA double-stranded breaks are repaired by error-prone mechanisms, e.g. non-homologous end joining, or homologous recombination when a repair template is provided (Aird et al. 2018; Huang and Puchta 2019). While significant advancements have been made in optimizing in-vivo delivery techniques and editing efficiencies (Luther et al. 2018; Demirci et al. 2022), efficient and reliable identification of CRISPR-edited organisms, or crispants, remains a time-consuming and tedious task.

Distinguishing between crispants and unedited individuals requires robust mutation detection methods. The most robust and widely used mutant screening methods are next-generation sequencing (NGS), whole-genome sequencing, and amplicon sequencing, along with Sanger sequencing. While genomic sequencing is highly accurate and offers a more comprehensive evaluation of genome-wide on-target and off-target-induced mutations, scaling it for high-throughput mutant screening is often impractical due to costs and complexity (Vonk et al. 2025). Single-molecule real-time sequencing and nanopore sequencing are emerging as promising alternatives to conventional NGS due to their ability to detect structural variations, long-range haplotypes, and epigenetic modifications in CRISPR-edited genomes (Ardui et al. 2018). Nonetheless, their current costs do not allow them to be applied for large-scale screening.

Alternatively, fluorescence-based Assays, which involve the use of fluorescently labeled dyes or proteins, can be applied *in vitro* for mutation detection, e.g. TaqMan probes, melting curve analysis, and real-time PCR (Huang et al. 2011). For example, digital PCR (dPCR) has emerged as a highly sensitive and precise alternative to detect rare mutations and low-abundance CRISPR-induced indels. Unlike traditional qPCR, dPCR partitions the sample into thousands of individual reactions, enabling absolute quantification of mutation events without the need for standard curves (Quan et al. 2018). Similarly, high-resolution melting analysis emerged as a cost-effective, post-PCR method for detecting small sequence variations, making it a useful complement to fluorescence-based assays (Wittwer et al. 2003). Another emerging method for mutation detection is amplicon-based CRISPR editing analysis using droplet-based microfluidics, which enhances sensitivity and throughput for detecting rare editing events (Zhao et al. 2023). Moreover, Cas9-induced indel detection by barcode sequencing is a novel approach that uses targeted deep sequencing to provide high-resolution profiles of CRISPR-mediated mutations and their distributions (Bell et al. 2014). These advancements contribute to improving the accuracy and scalability of mutant screening, particularly for large-scale or multiplexed CRISPR experiments. However, screening for simple CRISPR-induced mutations without *in vivo* modification, these new methods seem more labor intensive.

Among the most widely used cost-effective approaches for mutant screening are the T7 Endonuclease I (T7EI) assay and fragment analysis (FA). The T7EI assay is a mismatch detection method that identifies insertions and deletions (indels) at the target site by taking advantage of T7's ability to enzymatically cleave mismatched DNA heteroduplexes (Mashal et al. 1995; Youil et al. 1995). It is frequently employed due to its simplicity and rapid processing time. However, T7EI's sensitivity to heteroduplex formation can lead to false positives or inconsistent results (Babon et al. 1995; Mashal et al. 1995; Youil et al. 1995), particularly in organisms with complex genetic backgrounds (Sentmanat et al. 2018). Additionally, the assay struggles with detecting small indels or mutations present at low frequencies (in cell populations), making it less reliable for accurate mutation characterization (Vouillot et al. 2015).

FA is a precise and quantitative method for detecting Cas-induced mutations using capillary electrophoresis to distinguish DNA fragments based on size variations caused by indels. FA is advantageous for its quantitative nature and ability to detect CRISPR-induced mutations based on allele size variations of target loci, offering a higher resolution approach for identifying indels. The process begins with PCR amplification of the target locus using fluorescently labeled primers. The resulting amplicons are then separated by capillary electrophoresis, which accurately measures fragment sizes.

FA provides higher resolution than agarose-gel-based methods, enabling precise quantification of indel sizes. Unlike T7EI, it does not depend on heteroduplex formation, enhancing its reliability for detecting subtle mutations. Additionally, FA can differentiate between heterozygous and homozygous mutations, offering insights into mutation zygosity. However, the need for specialized equipment, such as a capillary-based genetic analyzer, restricts its use.

In this study, we compare the efficiency, accuracy, and limitations of the T7EIassay and FA for identifying CRISPR-induced mutations in the microcrustacean *Daphnia*. By evaluating their performance in detecting crispants, we aim to provide insights into which methods to choose for in vivo mutant screening, considering factors such as sensitivity, reproducibility, and practicality.

## Materials and methods

### *Daphnia* system, target genes for knockout, and screening strategies

*Daphnia magna* has been a model system for genomics, population, and evolutionary studies for several decades. With its whole genome sequenced and well annotated (Chaturvedi et al. 2023), it serves as a good system for undertaking CRISPR experiments. *D. magna*, as with other *Daphnia* species, has a cyclically parthenogenetic mode of reproduction where under favorable conditions females reproduce asexually making embryos without mating. When exposed to adverse conditions, female *Daphnia* switch to sexual reproduction where they produce recombinant offspring with males in the population. The parthenogenetic offspring are genetically identical to their mother. Previously, we showed that successfully edited females may produce offspring of a variable mutation profile (mosaicism) at the target sites (Xu et al. 2025). For our gene knockout experiments, we used the *D. magna* isolate LRV01, which has a high-quality genome assembly (Chaturvedi et al. 2023). Four genes, *DNMT3A*, *DNMT3B*, *PERIOD2*, and *DMRT1* were targeted for CRISPR/Cas9 knockout mutations (Table 1).

**Table 1.** CRISPR target sites and sgRNA target sequences.

Target gene	Gene ID	sgRNAs	Genomic location	Sequence
*DNMT3A*	Dmagna014665	*DNMT3A*-Mtase-sgRNA1	scaffold_4:1379821–1379843 (−)	GAAGAGGTGTACGAACTCAA
		*DNMT3A*-Mtase-sgRNA2	scaffold_4:1379724–1379746 (+)	GGTCGTGGTACTCCACGACA
*PERIOD2*	Dmagna002157	persgRNA2	scaffold_1:9233157–9233176 (−)	TCCCACGTATCCTCATTAAA
		persgRNA5	scaffold_1:9232817–9232836 (+)	CTCCTTTGTTGGATCCAGCT
*DNMT3B*	Dmagna014617	MA*DNMT3B*-sgRNA1	scaffold_4:1221965–1221987 (−)	CCCCTCTTTTCTATCTAGCCAAA
		MA*DNMT3B*-sgRNA2	scaffold_4:1221777–1221799 (−)	CCATCCGTTTTTTAACGGTGGTC
*DMRT1*	Dmagna000339	*DMRT1*dm	scaffold_1:1580546–1580565 (−)	TGTGCAAGAATCATGGCATC
		*DMRT1*-2	scaffold_1:1580730–1580749 (−)	TGGCCGAAATAGATGCCCTT

**Fig. 1.**
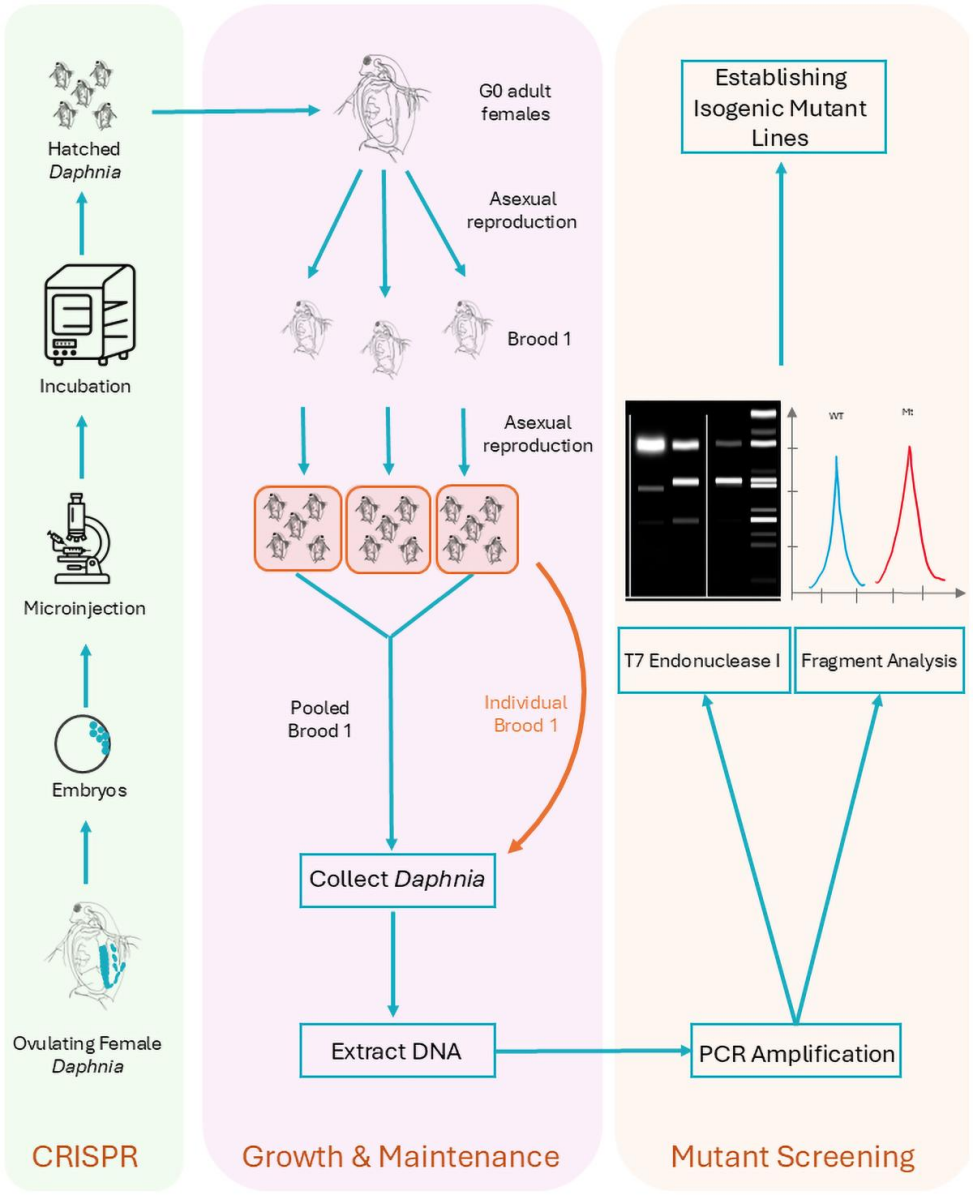
An illustration of the workflow of our CRISPR gene editing and mutant screening procedure.

We use FA to resolve subtle differences in target amplicon sizes caused by Cas-induced indels. This method allows us to distinguish between clean, single-signature edits and more complex mixtures, which are more prevalent in pooled samples. In parallel, T7 endonuclease assays are performed as a screen for heteroduplex formation, though we have observed that T7 can miss certain types of edits or give false positives, particularly in pooled brood 1 offspring (Fig. 1), consisting of multiple genotypes. Together, these methods help us piece together the mutation mosaics within brood 1 and determine whether edits were transmitted through the germline. For the DNMT3A gene, we whole-genome sequenced on 4 potential mutants with standard Illumina library preparation (∼6 million 150-bp paired-end reads for each mutant, ∼30× coverage). The raw reads were aligned to the *D. magna* LRV01 reference genome (Chaturvedi et al. 2023) using BWA-MEM (Li 2013), and raw read alignments were visually inspected for indels around the guide RNA target sites in the IGV viewer (Robinson et al. 2011). Genomic sequencing was not applied to the other genes because of the costs.

### SgRNA design and Cas9 delivery

For each target gene sequence, we used IDT's custom guide RNA design tool (https://www.idtdna.com/site/order/designtool/index/CRISPR_SEQUENCE) to identify optimal sgRNAs based on their on-target scores, which consider factors such as the surrounding nucleotide sequences, GC composition near the PAM site, and the presence of cytosines within the 20-nucleotide target region. Additionally, sgRNAs were selected based on their genomic location, prioritizing those within exons or protein domains to maximize the likelihood of functional disruptions. A subset of high-scoring crRNAs was then synthesized and assembled into ribonucleoprotein (RNP) complexes with Cas9 nuclease in vitro. The assembly involved first fusing each crRNA to a tracrRNA (supplied separately) to form a functional tracrRNA–crRNA hybrid (sgRNA), which was subsequently complexed with the Cas9 nuclease to generate a RNP complex.

The activity of the assembled RNP was tested in vitro by performing a digestion assay on the PCR amplicon containing the target site. Specifically, a PCR amplicon was designed with the target site positioned off-center, producing 2 fragments of unequal lengths, which upon digestion are easily distinguishable on an Agarose gel. For the digestion assay, 2 μL of PCR amplicon (>150 ng), 1 μL of nuclease reaction buffer, 1 μL of RNP, and 6 μL of water were mixed in a reaction tube. The mixture was then incubated at 37 °C for 1 h. Then, 1 μL of Proteinase K (20 mg/mL) was added and incubated at 56 °C for 10 min to deactivate and decouple the Cas9 enzyme. The digested product was run on a 1% agarose gel electrophoresis alongside an undigested wild-type (WT) control. Successful RNP cutting activity results in 2 distinct bands, corresponding to the cleaved fragments, whereas the WT sample shows a single band equal in length to the sum of the digested fragments.

### Microinjection for gene editing

We followed a previously described microinjection procedure for generating mutants (Xu et al. 2025). We selected about female daphniids with dark ovaries, indicating they were close to molting and ovulating (Fig. 1). Each was placed in COMBO medium (Kilham et al. 1998) with 60 mM sucrose and monitored for molting. We watched for ovulation, which starts 10 to 15 min post-molting. When ∼80% of embryos entered the brood chamber, the female was moved to ice-cold COMBO (1.5 °C) for 6 min to slow embryo development. We then dissected the embryos on an inverted Petri dish, aligned them along the edge, and kept a thin layer of COMBO over the embryos, which are ready for injection.

Injections were performed immediately after embryo preparation (Fig. 1). We backloaded 1 μL of RNP into microinjection needles using Eppendorf microloader tips (cat. no. 930001007). Each embryo was injected with 1 to 2 nL of RNP, released near the center using pressures between 100 and 220 hPa (background: 100 to 200 hPa) over 0.8 s. Post-injection, embryos were incubated in COMBO with 60 mM sucrose for ∼30 min at room temperature, then transferred to 60 mM sucrose COMBO in 96-well flat plates and cultured at room temperature. Hatching typically completes within ∼48 h.

### General strategy for screening mutants

After hatching, female neonates (G0 individuals) were separately cultured (Fig. 1). Offspring from the brood 1 of each G0 adult female were separated and individually cultured (Fig. 1). Once these brood 1 individuals asexually reproduced (usually took 7–10 d under regular feeding of algae), we pooled the offspring of all brood 1 animals derived from the same G0 mom for DNA extraction, genotyping using FA, and T7 assay for the target genes. Pooled samples showing signs of mutation were further analyzed by genotyping (T7 assay or FA) individual brood 1 isolates (Fig. 1) to identify which of the brood 1 individuals are mutants.

### T7 endonuclease I assay

The T7EI assay is commonly used to detect Cas-induced mutations by leveraging the enzyme's ability to recognize and cleave mismatched DNA sequences. The target genomic region is first amplified via PCR to produce double-stranded DNA fragments. These amplicons undergo denaturation and reannealing, promoting heteroduplex formation between WT and mutant DNA strands when mutations are present. T7EI selectively cleaves these heteroduplexes at mismatch sites, generating distinct DNA fragments that are subsequently analyzed by gel electrophoresis to determine the presence of mutations.

To perform the T7 digestion on pooled as well as individual samples, approximately 200 ng of PCR amplicon was combined with 2 μL of 10× NEBuffer 2 and molecular-grade water to a final volume of 19 μL. Hybridization was achieved by initial denaturation at 95 °C for 5 min, followed by annealing from 95 to 85 °C at a ramp rate of −2 °C/second and from 85 to 25 °C at −0.1 °C/second, with a final hold at 4 °C. Alternatively, after denaturation at 95 °C, the reaction can be allowed to cool gradually at room temperature, eliminating the need for programed ramp rates. After hybridization, 1 μL of T7 endonuclease (NEB catalog number M0302S, 10 units/µL) was added to the mix, which was then incubated at 37 °C for 20 min. The reaction was terminated by adding 1.5 μL of 0.25 M EDTA and then analyzed by agarose gel electrophoresis. The protocol was optimized using amplicons containing indels from an established scarlet gene knockout *Daphnia* mutant (Xu et al. 2025).

### Fragment analysis

We employed an M13 fluorescence-tagged PCR (Schuelke 2000) approach for FA. The forward primer was modified with an M13 tail sequence (CACGACGTTGTAAAACGAC) at its 5′ end (Table 2). The PCR reaction included the M13-tailed forward primer, a reverse primer (Table 2), and an M13 sequence labeled with one of the NED, PET, FAM, or VIC fluorescent dyes. The thermal cycling conditions were as follows: initial denaturation at 94 °C for 5 min, followed by 30 cycles of 94 °C for 30 s, 60 °C for 45 s, 72 °C for 30 s, and a final extension at 72 °C for 10 min. Two microliters of the PCR product was diluted and used for FA using LIZ ladder 600 (GeneScan™ 600 LIZ™, Catalog number 4408399) on an ABI 3130 Genetic Analyzer (Life Technologies) at DNA Core Facilities at University of Missouri, Columbia. We used the Osiris software 2.15.1 (NCBI) to score the size of alleles.

**Table 2.** Fragment analysis PCR primers.

Target gene	Primers	Primer sequence
*DNMT3A*	MADNMT3A-F	CACGACGTTGTAAAACGACGTTCCGACTGGGCCCTTAAA
	MADNMT3A-r	GATGACGTTGCGGATCGAAT
*PERIOD2*	Per2fw1	CACGACGTTGTAAAACGACGCACTCGACTGGAGGTGGTCCT
	Pero2rv	CCAACGACGTGGCCGAGATGAC
*DNMT3B*	MADNMT3B-F	CACGACGTTGTAAAACGACCGCAGTTCAGTGAATCCGA
	MADNMT3B-R	CAATTCTGTGCTTGTCCTATGG
*DMRT1*	DMRT1fw1	CACGACGTTGTAAAACGACTGTGCAGAATTCGGCCAGCGTT
	DMRT1rv1	CTCTGTCTTGTTGCTGGGCCCG

The forward primers include M13-tail (5′-CACGACGTTGTAAAACGAC-3′).

## Results and discussion

Although the T7EI assay is rapid and straightforward, its dependence on heteroduplex formation can result in variable detection efficiency. Small indels, particularly single-nucleotide insertions or deletions, may not create sufficient mismatches for enzyme recognition, leading to false negatives. Additionally, T7EI's activity is influenced by sequence context, reducing its reliability for detecting certain mutations. These limitations are generally acknowledged by the manufacturer, noting that the enzyme has low sensitivity for mismatches of unknown composition, single-base pair mismatches, and may require titration for optimal cleavage. These constraints pose challenges when screening for unknown types of mutations that are potentially induced by the Cas enzyme.

### Case study #1—*DNMT3A* gene knockout

Nine samples of brood 1 for the *DNMT3A* gene knockout were initially screened for Cas-induced mutations using the T7EI assay. Out of the 9, 4 samples tested positive (Table 3), showing additional bands indicative of potential mutations. To verify these results, whole genome sequencing instead of FA analysis was subsequently performed on the 4 T7-positive samples. Interestingly, WGS did not detect any mutations in these samples (Table 3). This outcome suggests the occurrence of false positives in the T7EI assay, highlighting its limitations in mutation detection accuracy.

**Table 3.** Mutant screening of 3A1-3A9 for the DNMT3A gene.

DNMT3A	WGS	T7EI
3A1	N/A	Negative
3A2	N/A	Negative
3A3	N/A	Negative
3A4	N/A	Negative
3A5	Negative	Positive
3A6	Negative	Positive
3A7	N/A	Negative
3A8	Negative	Positive
3A9	Negative	Positive

Mutant screening was done using the T7 endonuclease assay and whole-genome sequencing. Positive refers to a mutation detected and negative refers to no mutation.

### Case study #2—PERIOD2 gene knockout

**Fig. 2.**
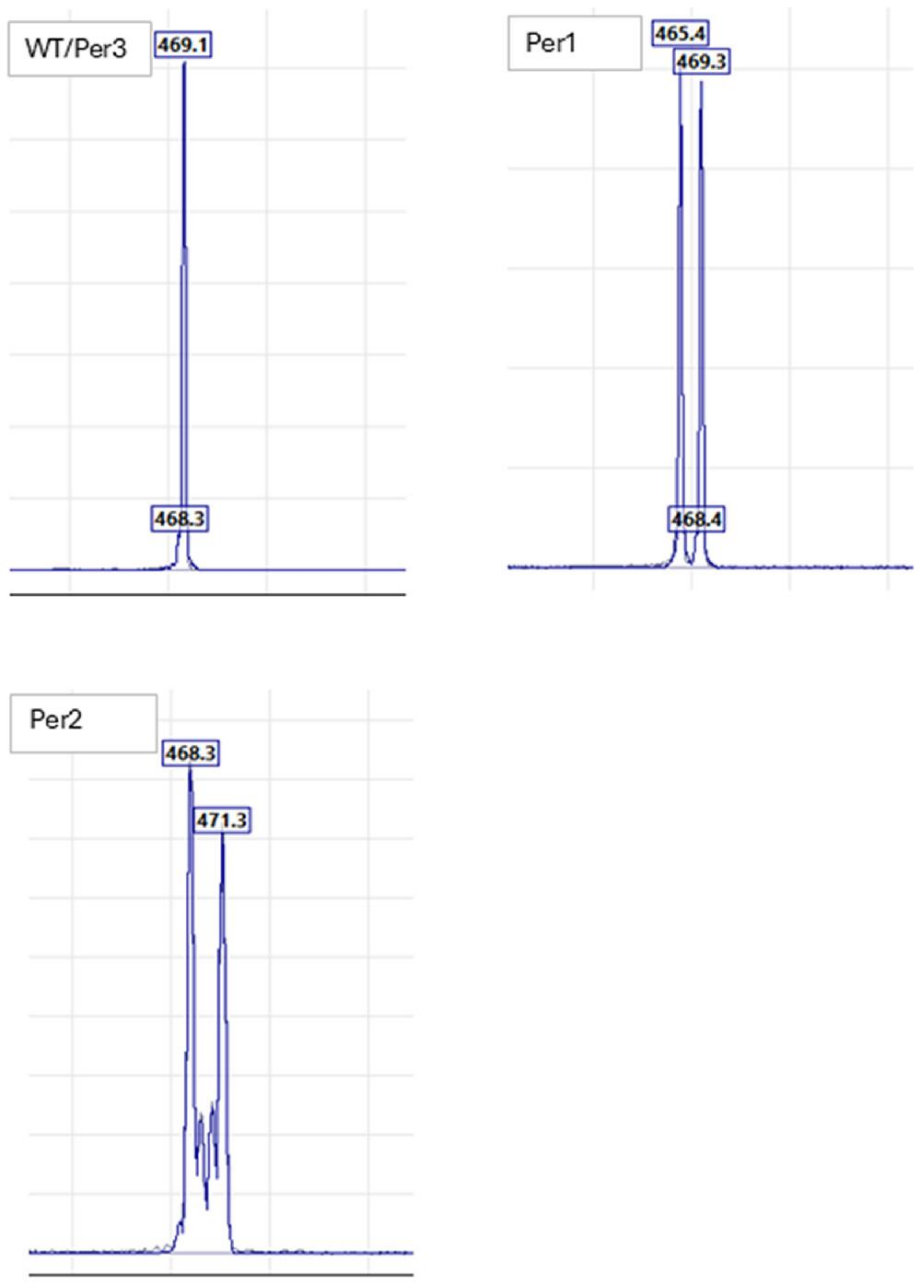
Fragment analysis of the PERIOD*2* gene. Wild-type genotype is homozygous (469/469). The 2 knockout mutants Per1 and Per2 were heterozygous with new mutated alleles.

A total of 25 brood 1 samples were screened for CRISPR-induced mutations in the Per2 (*PERIOD2*) gene using T7EI. Of these, 3 tested positive, suggesting potential mutations. To validate these results, FA was performed on the T7EI-positive samples (Table 4). FA confirmed mutations in only 2 samples (Per1 and Per2, Fig. 2), revealing 1 false positive (Per3) from the T7EI assay.

**Table 4.** Mutant screening of 3 mutants for the PERIOD2 gene.

Samples	FA	T7EI
Per1	Positive	Positive
Per2	Positive	Positive
Per3	Negative	Positive

Two mutants were supported by both the T7EI assay and fragment analysis. Positive refers to a mutation detected and negative refers to no mutation.

This discrepancy highlights the limitations of T7EI, which can produce false positives due to its reliance on heteroduplex formation. In contrast, FA provided more accurate detection based on amplicon lengths. These findings underscore the importance of validating T7EI results with a more accurate method like FA for reliable mutation screening.

### Case study #3—*DNMT3B* gene knockout

**Fig. 3.**
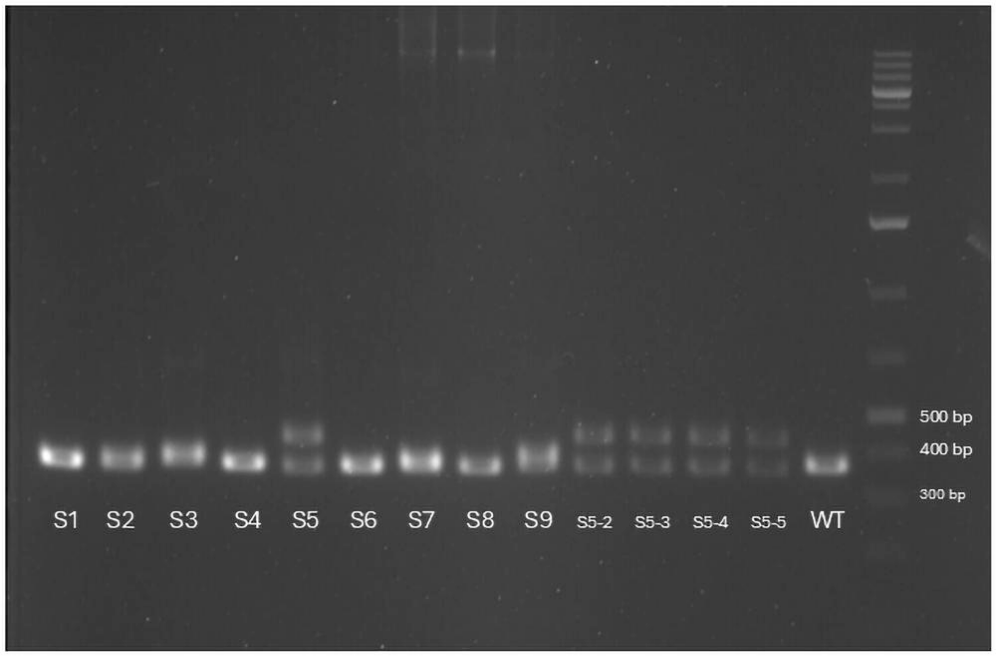
PCR amplification for the DNMT3B gene of S1 to S9, and individual brood 1 genotypes from S5 mom.

**Fig. 4.**
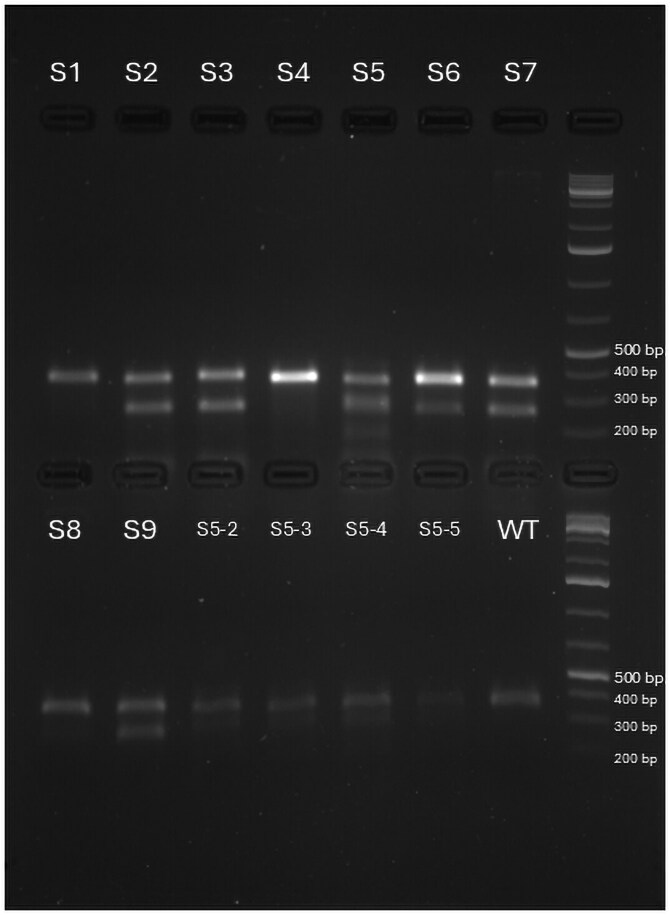
T7 endonuclease assay on DNMT3B mutant screening. S2, S3, S5, S6, S7, and S9 are positive for mutation. S5-2 to S5-5 are the brood 1 offspring of S5. S1 to S9 are pooled brood 1 from their respective parent.

A total of 9 pooled samples, each representing a brood 1 offspring genotype from knockout experiments targeting the *DNMT3B* gene, were screened for mutations. For each sample, 3 to 5 brood 1 individuals were pooled for FA. Out of nine, 1 sample (S5) showed a distinct PCR amplicon profile (Fig. 3) on agarose gel, including a WT-like band and an additional higher molecular weight band, suggesting a potential insertion (later verified with FA). The remaining 8 samples showed WT-like bands and were further investigated using both T7 and Endonuclease I (T&EI) and FA. FA revealed mutations in 6 samples: S2, S3, S5, S6, S7, and S9 (Table 5). T7EI cleavage on the same set also detected mutations in these same 6 samples (Fig. 4). In this case, T7EI and FA consistently identified the same set of positive samples, supporting their agreement in detecting CRISPR-induced mutations.

**Table 5.** Mutant screening of S1–S9 for the DNMT3B gene using fragment analysis and T7 endonuclease assay.

DNMT3B	Fragment analysis	T7EI
S1	Negative	Negative
S2	Positive	Positive
S3	Positive	Positive
S4	Negative	Negative
S5	Positive	Positive
S6	Positive	Positive
S7	Positive	Positive
S8	Negative	Negative
S9	Positive	Positive

Positive refers to a mutation detected and negative refers to no mutation.

### Case study #4—*DMRT1* gene knockout

**Fig. 5.**
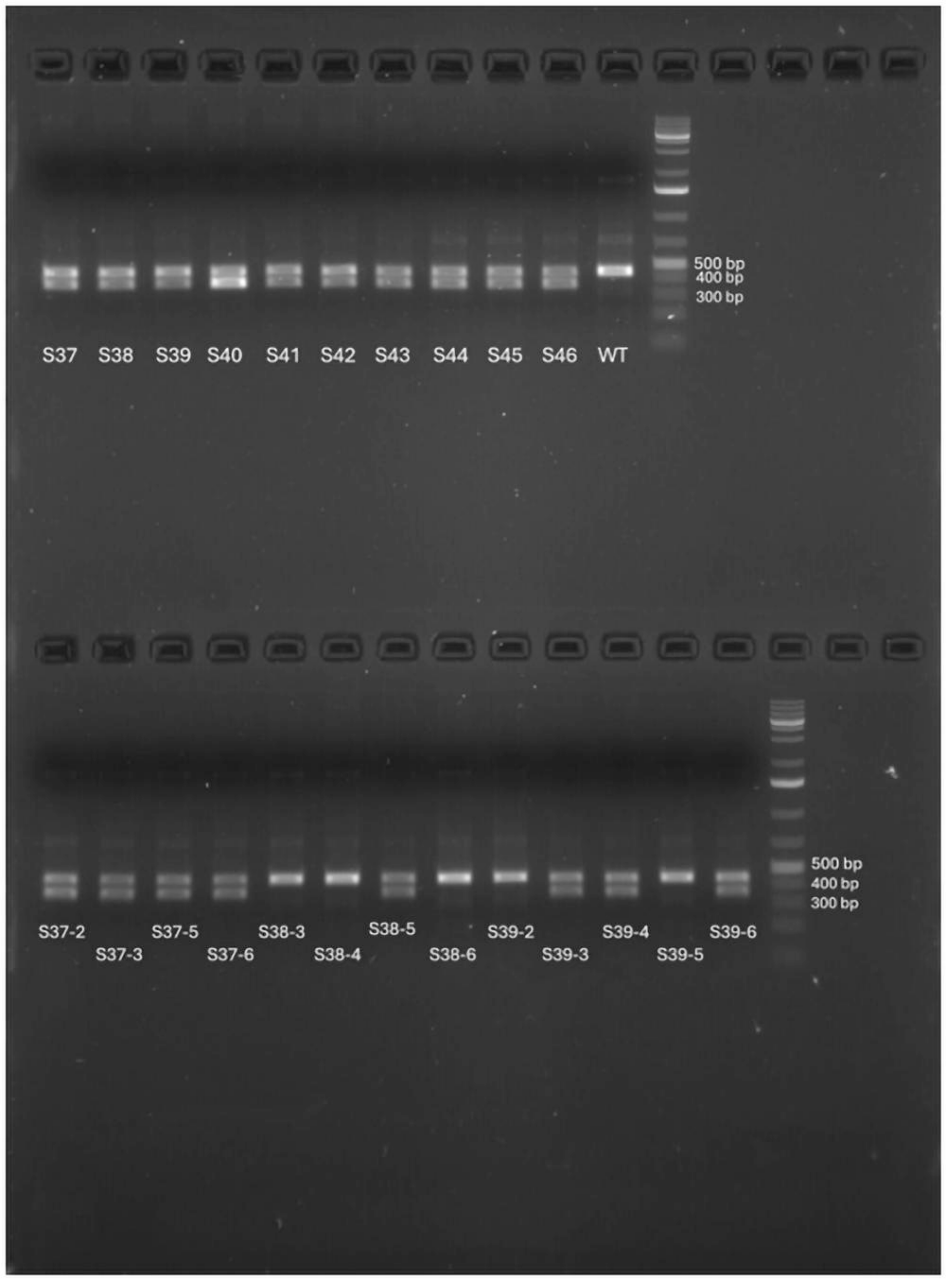
T7 on the pooled DMRT1 gene for samples S37 to S46, all are positive for the T7 assay. T7 assay on individual brood 1 genotype from S37, S38, and S39. S38-3, S38-4, S38-6, and S39-5 showed no mutations, which is consistent with the FA analysis result. T7 assay indicated no mutation for s39-2, conflicting the FA analysis result.

A total of 10 pooled samples, each representing a brood 1 offspring experiment knocking out the *DMRT1* gene, were screened for mutations. FA detected mutations in 3 samples (Table 6). In contrast, T7EI cleavage assays showed positive results for all 10 samples, suggesting that all harbored some form of mutation (Fig. 5).

**Table 6.** Mutant screening of S37-S36 for DMRT1 gene using fragment analysis and T7 endonuclease assay.

DMRT1	Fragment analysis	T7EI
S37	Positive	Positive
S38	Positive	Positive
S39	Positive	Positive
S40	Negative	Positive
S41	Negative	Positive
S42	Negative	Positive
S43	Negative	Positive
S44	Negative	Positive
S45	Negative	Positive
S46	Negative	Positive

Positive refers to a mutation detected and negative refers to no mutation.

However, upon careful comparison with FA results, T7EI falsely identified 7 pooled samples as containing mutants. When T7EI was subsequently performed on individual (non-pooled) brood 1 samples, it was able to correctly show negative results for those that did not contain mutations (Fig. 5, Table 7), highlights the limitation of T7 assays.

**Table 7.** Mutant screening of individual brood 1 from S37, S38, and S39 for the DMRT1 gene using fragment analysis and T7 endonuclease assay.

DMRT1	Fragment Analysis	T7EI
S37-2	Positive	Positive
S37-3	Positive	Positive
S37-5	Positive	Positive
S37-6	Positive	Positive
S38-3	Negative	Negative
S38-4	Negative	Negative
S38-5	Positive	Positive
S38-6	Negative	Negative
S39-2	Positive	Negative
S39-3	Positive	Positive
S39-4	Positive	Positive
S39-5	Negative	Negative
S39-6	Positive	Positive

Positive refers to a mutation detected and negative refers to no mutation.

### Sensitivity and accuracy

When comparing the 2 methods, FA demonstrated superior sensitivity in detecting small indels that were often missed by T7EI. The ability of capillary electrophoresis to resolve single-base pair differences provided an advantage over the enzymatic cleavage method, which relies on imperfect heteroduplex recognition. In contrast, T7EI was found to be more effective for screening large deletions but struggled with smaller mutations (see manual for Cat. No. M0302, New England Biolabs). The T7 assay also shows different efficiency with different mismatches (Guan et al. 2004). This limitation makes FA the preferred method for applications requiring high-resolution indel analysis.

### Reproducibility and practical considerations

Reliability and reproducibility are critical for mutation detection. FA showed high reproducibility across multiple independent experiments, with minimal variation in fragment sizing and less optimization required. T7EI, however, exhibited inconsistent cleavage efficiency depending on sequence context, leading to variability in results. Additionally, the requirement for gel electrophoresis in T7EI introduces subjective interpretation, whereas FA provides objective numerical data for sizing of the alleles.

From a practical standpoint, T7EI remains an attractive option for laboratories with limited resources due to its lower cost and ease of implementation. It allows for rapid screening of large sample sets without the need for specialized instrumentation. On the other hand, FA, while requiring advanced equipment, provides more precise and reliable mutation characterization, making it an excellent choice for confirmatory analysis.

While sequencing remains the benchmark for mutation confirmation due to its ability to provide exact nucleotide changes (Knott and Doudna 2018), it is often impractical for handling large sample sets due to cost and time constraints. In such cases, FA and T7EI serve as feasible alternatives for high-throughput screening. FA, with its high resolution, offers a reliable method for detecting small indels, whereas T7EI provides a rapid and cost-effective option for initial screening although with higher chances of capturing falsepositives. The choice between these methods depends on the specific needs of the study, with sequencing being the ideal validation tool when resources permit.

The T7E1 mismatch detection assay is widely utilized for assessing CRISPR-Cas9 editing efficiency. However, studies comparing T7E1 results with targeted NGS have demonstrated discrepancies, with T7E1 often failing to accurately reflect the true editing efficiency (Sentmanat et al. 2018). Moreover, other alternative methods, such as Tracking of Indels by Decomposition (Brinkman et al. 2014) and Indel Detection by Amplicon Analysis (Yang et al. 2015) have shown greater concordance with NGS data. These findings highlight the limitations of T7E1 in detecting certain mutation profiles and suggest that while useful for initial screening, it may not always provide reliable editing estimates.

## Conclusion

In this study, we compared 2 commonly used methods for mutant screening: the T7EI assay and FA. Our findings highlight the strengths and limitations of each approach. T7EI is a cost-effective and rapid screening tool, but it lacks sensitivity for detecting small mutations and is prone to false negatives. FA, while requiring specialized equipment, provides superior accuracy and resolution, making it the preferred choice for precise mutation analysis.

Ultimately, the choice of method depends on the specific requirements of a study. Laboratories seeking a quick and affordable screening method may benefit from T7EI, whereas those requiring high-resolution, higher sample quantities, and a demand for quantitative mutation detection should consider FA. By understanding the trade-offs between these techniques, researchers can select the most appropriate tool for their CRISPR screening needs, particularly in whole-organism studies like our study conducted in *Daphnia*. We conclude that the T7EI assay requires frequent optimizations based on amplicon length and composition, and types of mutation to detect (single base mismatches to large indels, heterozygous and homozygous mutations). In contrast, FA needs very little optimization except for optimizing the annealing temperature in PCR to reduce spurious amplifications. FA also provides single-base pair resolution when it comes to identifying mutant alleles.

## Data Availability

The authors affirm that all data necessary for confirming the conclusions of the article are present within the article, figures, and tables.
